# Hospitalization and Credit Scores Among Medicaid Beneficiaries in Louisiana

**DOI:** 10.1001/jamahealthforum.2025.1570

**Published:** 2025-06-06

**Authors:** Brigham Walker, Gael Compta, Alexander Siebert, Andrew Anderson, Kevin Callison, Chima D. Ndumele, Jacob Wallace

**Affiliations:** 1 Tulane University, New Orleans, Louisiana; 2 Yale University, New Haven, Connecticut; 3 Johns Hopkins University, Baltimore, Maryland

## Abstract

This case-control study evaluates the impact of a hospitalization on credit scores for Medicaid beneficiaries in Louisiana stratified by sex, race, and ethnicity.

## Introduction

Hospitalizations can impose financial hardships on families,^1,2^ contributing to lower credit scores^3^ and reducing access to credit.^1^ Non-Hispanic Black and Hispanic patients may struggle more with hospitalization costs due to lower wealth compared with non-Hispanic White patients.^4^

We evaluated the impact of a hospitalization on credit scores for Medicaid beneficiaries in Louisiana, examining effects by sex, race, and ethnicity. Louisiana is a setting well-suited for this research given that it is a Medicaid expansion state, has relatively high levels of personal debt (which inform credit scores), and has a relatively diverse population.

## Methods

Health care claims data from the Louisiana Medicaid program were used to identify beneficiaries with any nonpregnancy-related hospitalization in 2018. This time period avoids potentially confounding effects due to the 2016 Medicaid expansion and the COVID-19 pandemic in 2020. The comparison group includes Medicaid beneficiaries who were not hospitalized or pregnant, to whom we randomly assigned a “pseudohospitalization” date in 2018 allowing for a contemporaneous comparison.

Beneficiaries in both groups were continuously enrolled for 12 months before and after their respective hospitalization month. The claims data were then linked to anonymized credit report data from one of the national consumer credit reporting bureaus. The key outcome variables were credit score (a continuous variable) and the probability of having a poor credit score (below 580). Data were aggregated to the year-quarter level. The Louisiana Department of Health and Tulane University institutional review boards approved this study and waived informed consent. This study followed the Strengthening the Reporting of Observational Studies in Epidemiology (STROBE) reporting guidelines.

### Statistical Analysis

We used a difference-in-differences model where the estimates varied over time relative to the shock date. This allowed us to evaluate (1) whether the 2 groups trended similarly in the preperiod and (2) the potential timing of the hospitalization effects. Baseline observed characteristics between treatment and control groups were balanced using a maximum entropy reweighting scheme to align means and variances.^5,6^ Finally, to evaluate potential heterogeneous effects, we stratified analyses separately for Hispanic, non-Hispanic Black, and non-Hispanic White populations; and for male individuals and female individuals. The analysis took place between 2024 and 2025.

## Results

**Figure 1.**
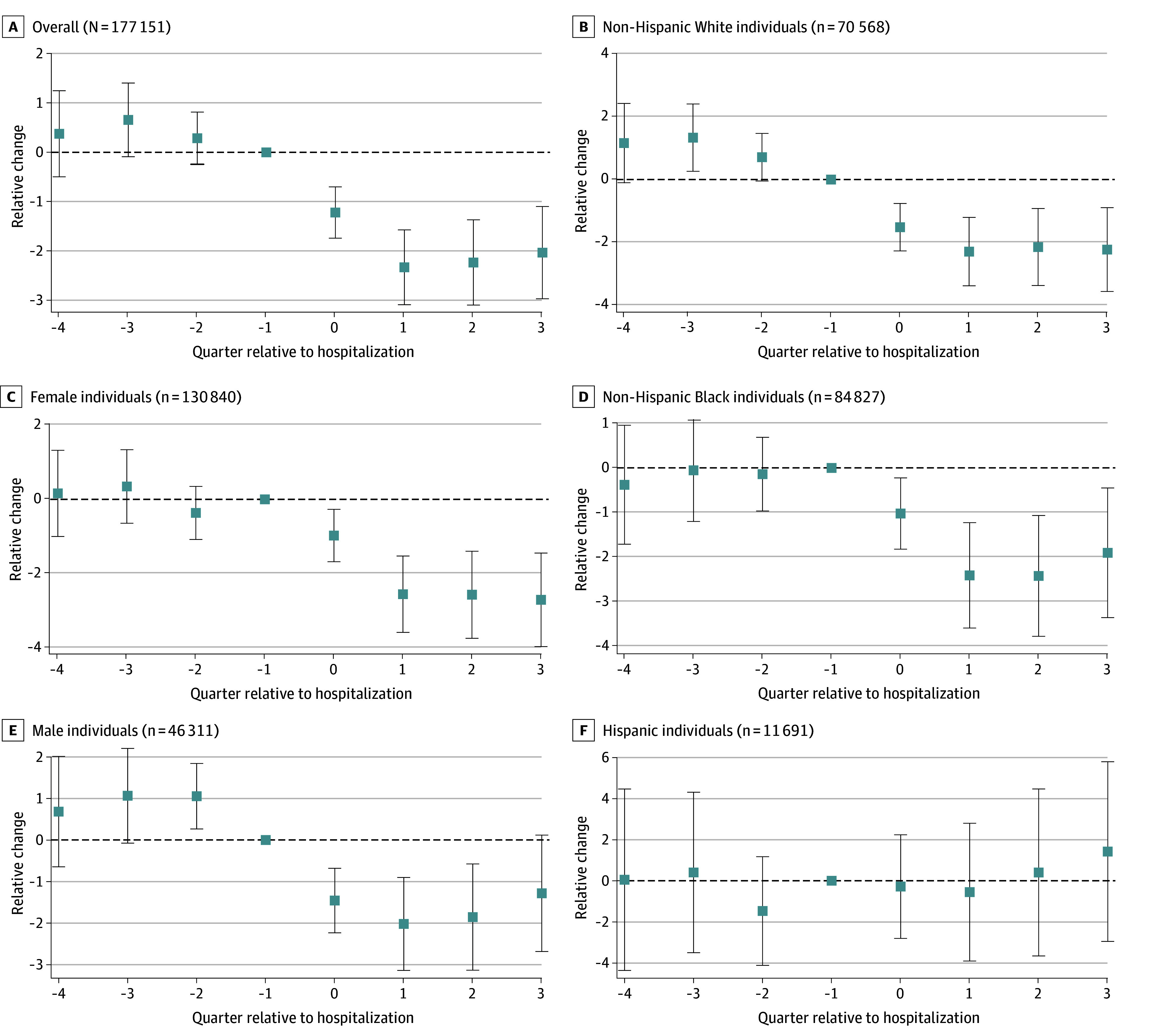
Association of Hospitalization With Credit Score Each individual had 8 observations. Hospitalizations occurred in 2018. For perspective, approximately 2% had a myocardial infarction and approximately 1% had a stroke. Quarter 0 is the quarter in which the hospitalization occurred (and it occurred in the first month of that quarter). Credit score values are monthly average values within that quarter. The boxes indicate point estimates and the whiskers indicate the 95% CIs.

Relative reductions in credit scores were apparent within the first quarter following hospitalization and were largely sustained for all groups (Figure 1A-E) except for Hispanic beneficiaries (Figure 1). These reductions leveled off in the second quarter following a hospitalization in which beneficiaries overall saw an average decrease of just over 2 points. Parallel trends generally held for all groups except non-Hispanic White and male beneficiaries (Figure 1).

**Figure 2.**
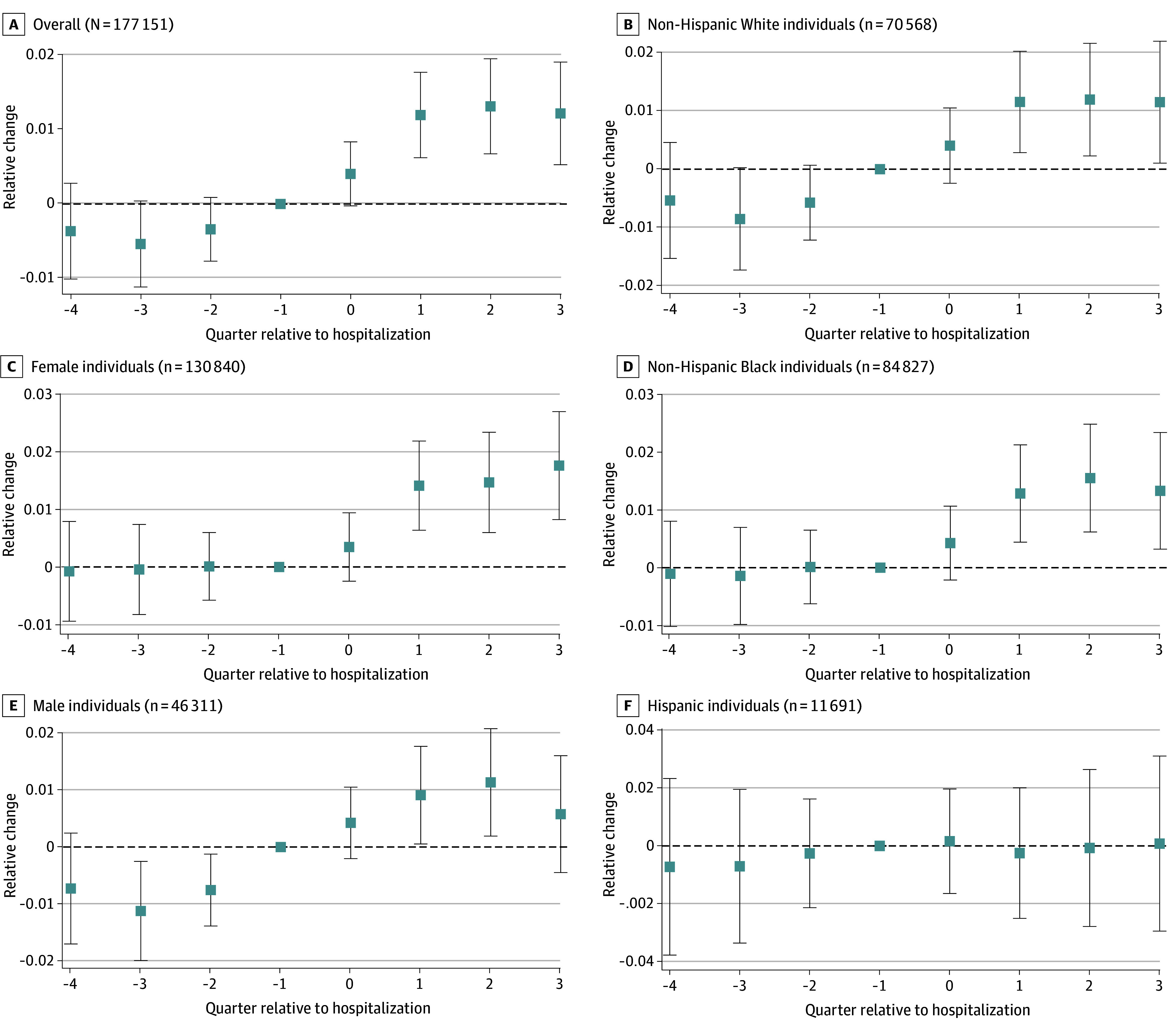
Association of Hospitalization With the Probability of Having Bad Credit (<580) Each individual had 8 observations. Hospitalizations occurred in 2018. For perspective, approximately 2% had a myocardial infarction and approximately 1% had a stroke. Quarter 0 is the quarter in which the hospitalization occurred (and it occurred in the first month of that quarter). The boxes indicate point estimates and the whiskers indicate the 95% CIs.

Similarly, all groups except for Hispanic beneficiaries saw increases in the probability of having poor credit, which peaked in the third quarter following a hospitalization at just over 1 percentage point (Figure 2A).

## Discussion

These results suggest that, despite facing no cost-sharing, Medicaid beneficiaries who were hospitalized faced modest negative consequences to their credit scores and may require more financial support to cope with a hospitalization. The source of these credit effects requires more study, but could be due to disruptions in employment. Given that baseline credit scores were close to the threshold between poor and fair, these negative consequences translate to small—but potentially consequential—increases in the probability of having poor credit.

Although there are several limitations to this case-control study, such as the single-state setting that may not generalize nationally, instances of nonparallel pretrends for non-Hispanic White and male beneficiaries, a focus on Medicaid beneficiaries only, and possibly underestimated effects because the first period after hospitalization likely includes some days prior to hospitalization, the results consistently showed worsening credit measures across sex and heterogeneous effects between Hispanic and non-Hispanic groups following a hospitalization.

## Notes

Supplement. Data Sharing Statement
