# Supplementary material for: Hospitalization and Credit Scores Among Medicaid Beneficiaries in Louisiana
Source: JAMA Health Forum. 2025 Jun 6;6(6):e251570. doi: 10.1001/jamahealthforum.2025.1570 (PMC12144616; doi:10.1001/jamahealthforum.2025.1570)
Supplement: Supplement. — Data Sharing Statement [file jamahealthforum-e251570-s001.pdf]

Data Sharing Statement

Walker. Hospitalization and Credit Scores Among Medicaid Beneficiaries in Louisiana. *JAMA Health Forum*. Published June 06, 2025. doi:10.1001/jamahealthforum.2025.1570

Data
Data available: No

Additional Information
Explanation for why data not available: Data are private
